# Barriers in Referring Neonatal Patients to Perinatal Palliative Care: A French Multicenter Survey

**DOI:** 10.1371/journal.pone.0126861

**Published:** 2015-05-15

**Authors:** Barthélémy Tosello, Lionel Dany, Pierre Bétrémieux, Pierre Le Coz, Pascal Auquier, Catherine Gire, Marie-Ange Einaudi

**Affiliations:** 1 Aix-Marseille University/EFS/CNRS, UMR 7268 ADÉS, Espace Éthique Méditerranéen, Hospital La Timone, 13005, Marseille, France; 2 Assistance Publique-Hôpitaux de Marseille, Hospital Nord, Department of Neonatology, 13015, Marseille, France; 3 Aix-Marseille University, LPS EA 849, 13621, Aix-en-Provence, France; 4 Assistance Publique-Hôpitaux de Marseille, Hospital La Timone, Department of Oncology, 13005, Marseille, France; 5 University Hospital Center, Hospital Sud, Department of Neonatology, Multidisciplinary Center for Prenatal Diagnosis, 35000, Rennes, France; 6 Aix-Marseille University, EA 3279, Self-Perceived Health Assessment Research Unit, 13005, Marseille, France; University of Rennes-1, FRANCE

## Abstract

**Background:**

When an incurable fetal condition is detected, some women (or couples) would rather choose to continue with the pregnancy than opt for termination of pregnancy for medical reasons, which, in France, can be performed until full term. Such situations are frequently occurring and sometimes leading to the implementation of neonatal palliative care. The objectives of this study were to evaluate the practices of perinatal care french professionals in this context; to identify the potential obstacles that might interfere with the provision of an appropiate neonatal palliative care; and, from an opposite perspective, to determine the criteria that led, in some cases, to offer this type of care for prenatally diagnosed lethal abnormality.

**Methods:**

We used an email survey sent to 434 maternal-fetal medicine specialists (MFMs) and fetal care pediatric specialists (FCPs) at 48 multidisciplinary centers for prenatal diagnosis (MCPD).

**Results:**

Forty-two multidisciplinary centers for prenatal diagnosis (87.5%) took part. In total, 102 MFMs and 112 FCPs completed the survey, yielding response rate of 49.3%. One quarter of professionals (26.2%) estimated that over 20% of fetal pathologies presenting in MCPD could correspond to a diagnosis categorized as lethal (FCPs versus MFMs: 24% vs 17.2%, p = 0.04). The mean proportion of fetal abnormalities eligible for palliative care at birth was estimated at 19.30% (± 2.4) (FCPs versus MFMs: 23.4% vs 15.2%, p = 0.029). The degree of diagnostic certainty appears to be the most influencing factor (98.1%, n = 207) in the information provided to the pregnant woman with regard to potential neonatal palliative care. The vast majority of professionals, 92.5%, supported considering the practice of palliative care as a regular option to propose antenatally.

**Conclusions:**

Our study reveals the clear need for training perinatal professionals in perinatal palliative care and for the standardization of practices in this field.

## Introduction

Inherited major structural or genetic abnormalities affect around 3% of births [1,2] and are one of the primary causes of infant morbidity and mortality. Some of these conditions are incompatible with long-term survival after birth [3]. It is not always possible to predict lethality, even in a fetus presenting severe abnormalities, and it is rare to have cases of long-term survivors with a severe disability [3]. French law [4] stipulates that, at the pregnant woman's request, termination of pregnancy for medical reasons (TOP) may be performed until full term provided that two doctors from a multidisciplinary team confirm either that continuing with the pregnancy would put the mother’s health at serious risk, or that there is a high probability that the unborn child will have a particularly serious condition already recognized as incurable at the time of antenatal diagnosis. In this situation, some women decide to continue with the pregnancy [5].

There has been much work published on strategies for managing pregnancies with lethal fetal abnormalities based on the expert opinions [6–11]. While many "recommendations" advise what perinatal professionals should do [6], there is only little data on what professionals actually do in practice. It is therefore important that multidisciplinary teams analyze and evaluate their professional practices. Our recent qualitative research [12] revealed the place accorded to the concept of neonatal palliative care and confirmed that these practices take into account multiple factors (professional, information-related, human and ethical), which question the fundamentality of multidisciplinary approach. However, consistency between antenatal and postnatal care teams, involving joint discussion, appears essential in order to guarantee continuity of care and respect the process of parenthood [11]. In order to be able to standardize the practices at a national level, we needed to review the opinions and practices at multidisciplinary centers for prenatal diagnosis (MCPD). Based on these findings, we put our primary objective to evaluate the practices of perinatal care professionals in France in the domain of palliative care. As secondary objective, we sought to identify the potential barriers to the routine offering of neonatal palliative care; and, from an opposite perspective, to determine the criteria that contributed to offer this type of care in cases of prenatally diagnosed lethal abnormality.

## Materials and Methods

The research team designed the questionnaire and conducted the survey using electronic reminders (three reminders) between April 2012 and December 2012. Ethical approval was obtained from the University Hospital ethics committee of Marseille, France; and the French Advisory Committee for Information Processing in Health Research expressed a favorable opinion of the performance of this research (reference number: 12.154).

### Sample and List Development

We used a database provided by MCPD (a master file available via the website of each center) to conduct a national survey concerning all perinatal professionals working in MCPD in France. We began by identifying all fetal care and fetal treatment centers from national listings of related research centers, professional organizations, and children’s hospitals. We searched in the websites and physician directories to reach the pediatric subspecialists providing fetal diagnostic or treatment services in those centers. We made additional Internet searches and telephone calls to the centers for confirmation. The resulting file contained 434 email addresses from 48 MCPD. Two groups of perinatal professionals were identified: maternal fetal medicine specialists (MFMs) in obstetric gynecology, midwifery or psychology; and fetal care pediatric specialists (FCPs) in neonatology, pediatric intensive care or genetics.

### Survey Instrument Development

We developed the questionnaire following a qualitative study using focus groups from two French multidisciplinary centers for prenatal diagnosis [12]. The final survey instrument consisted of 12 pages, comprising 3 parts: (1) Sociodemographic and professional characteristics (i.e. age, gender, professional status, seniority in the profession and in MCPD); (2) Opinions on lethal fetal abnormalities, neonatal palliative care and their management (i.e. definitions and perceptions of neonatal palliative care: definition of lethal fetal abnormality (LFA), perceived difficulties concerning LFA diagnosis and prognostic, perceived proportion of LFA, existence and usefulness of a LFA list, training in palliative care, opinions toward palliative care in neonatal period); and (3) Practices related to lethal fetal abnormalities and neonatal palliative care (i.e. criteria influencing management of LFA, criteria influencing antenatal communication of information about palliative care). A final draft questionnaire was pretested on 5 physicians (neonatologists and obstetricians) from the target eligible group to determine ease of understanding and completion time. Self-administered pretests take approximately 20 minutes. The questionnaire was posted online (via a private website created for the study) on April 2012 for a period of 9 months. Three email reminders were sent during this period. Completed questionnaires were automatically sent to an email address created for the study. Anonymity was strictly respected.

### Statistical Analysis

Descriptive data were expressed as numbers and percentages, supplemented with the corresponding means and standard deviations (SD) when applicable. Comparison of variables between the two groups of specialists was made using the chi-square test or analysis of variance, depending on the type of variable. Non-parametric tests were applied for variables with non-normal distribution. The threshold of significance of the tests was fixed at 5%. Statistical analyses were carried out using SPSS version 18 software.

## Results

Forty-two MCPD (87.5%) took part in the study. In total, 102 MFMs and 112 FCPs completed the survey, yielding response rate of 49.3%, and a mean of 5 professionals from each MCPD. Table 1 shows the characteristics of the professionals.

**Table 1:** Sociodemographic characteristics of the participating professionals.

Characteristics	Total n = 214	MFMs (%), n = 102	FCPs (%), n = 112	*P* value
**Age, y**				
Mean ± SD	41.9 ± 11.7	43.2 ± 11.0	40.6 ± 12.2	NS
**Sex**				
Male	65 (30.4)	23 (22.5)	42 (37.5)	0.018
Female	149 (69.6)	79 (77.5)	70 (62.5)	
**Role**				
Obstetric Gynecologist	61 (28.5)	61 (59.8)	―	―
Midwife	36 (16.8)	36 (35.2)	―	―
Psychologist	5 (10.6)	5 (4.9)	―	―
Neonatologist	58 (27.1)	―	58 (51.8)	―
Pediatric and neonatal intensive care specialist	40 (18.6)	―	40 (35.7)	―
Geneticist	8 (3.7)	―	8 (7.1)	―
Other	6 (2.8)	―	6 (2.8)	―
**Professional Experience, years**				
< 10 y	89 (41.6)	39 (38.6)	50 (45.5)	NS
≥ 10 y	122 (57.0)	62 (61.4)	60 (54.5)	
**Total Professional Experience in MCPD, months**				
< 60 m	62 (36.7)	20 (24.4)	42 (48.3)	0.001
≥ 60 m	107 (63.3)	62 (75.6)	45 (51.7)	

MFMs: Maternal Fetal Medicine specialists; FCPs: Fetal Care Pediatric specialists; MCPD: Multidisciplinary Center for Prenatal Diagnosis.

### Clinical Experiences and Professional Attitudes towards Lethal Fetal Conditions

The majority of professionals (97.7%, n = 209) considered the purpose of antenatal diagnosis as part of a curative care plan for the fetus, an attitude particularly prevalent among MFMs. Professionals generally considered that the MCPD has a duty to provide women with advices and information (96.7%, n = 207). Table 2 shows and compares the definitions and perceptions of lethal fetal abnormality (LFA) as reported by perinatal professionals. In total, 26.2% (n = 46) of professionals estimated that over 20% of fetal abnormalities presenting in MCPD could be categorized as lethal. The mean estimated proportion of LFA was more elevated among FCPs than MFMs (MFMs vs FCPs, 17.2% and 24.0% respectively (p = 0.04)). The procedures to be followed when managing lethal fetal abnormalities (trisomy 18, severe or syndromic diaphragmatic hernia, anencephaly, severe hydrocephalus, bilateral renal agenesis) were varied. More than half of the professionals considered TOP to be the appropriate course of action for trisomy 18 (50.1%, n = 112) and anencephaly (55.6%, n = 119). Neonatal palliative care was considered the appropriate strategy for severe hydrocephalus (11.5%, n = 24) and bilateral renal agenesis (20.1%, n = 43).

**Table 2:** Definitions and perceptions of lethal fetal abnormality (LFA) reported by perinatal professionals.

	MFMs, n%	FCPs, n%	*P* value
How can we describe LFA? *			
Short life expectancy of the newborn	90 (89.1)	103 (94.5)	NS
Certainty of neonatal death	83 (83.0)	92 (84.4)	NS
No reasonable therapeutic options available	77 (77.0)	90 (82.6)	NS
**Diagnosis and prognosis of LFA:**			
Difficulty in diagnosing LFA	73 (73.7)	94 (85.5)	0.035
Prognostic uncertainty of LFA	29 (29.0)	37 (33.9)	NS
**Proportion of fetal abnormalities presenting in MCPD corresponding to LFA:**			
(mean ±SD)	17.2 ± 19.3	24 ± 23.5	0.040
**Does your MCPD have an LFA list?**			
No	63 (62.4)	43 (38.7)	< 0.001
Don't know	33 (32.7)	65 (58.6)	
**Usefulness of an LFA list in practice at the MCPD**	49 (49.0)	65 (58.6)	NS

*only 3 options were given to the professionals.

### Neonatal Palliative Care Procedures

Nearly half of the professionals surveyed (47.8%, n = 98) reported that they had received practical training in palliative care—this being more common among FCPs (59.6% vs 34.4%, p < 0.001)—while one quarter (n = 54) had received theoretical formation.

Palliative care, defined as “*active and continuous care provided by a multidisciplinary team in an institution or at home*. *It is intended to relieve pain*, *reduce mental suffering*, *preserve the patient's dignity and support their family and friends*" [13], was provided as such by nearly 80% (n = 157) of the professionals and particularly by FCPs (MFMs: 66.7% vs FCPs: 90%, p < 0.001). The mean proportion of fetal abnormalities eligible for palliative care was estimated at 19.3% (± 2.4), with this figure significantly differing between MFMs and FCPs (15.2% vs 23.4% respectively, p = 0.029). Of the professionals surveyed, 20.5% (n = 35) estimated that over 20% of fetal abnormalities could result in the provision of palliative care after birth. Table 3 reports the definitions and perceptions of neonatal palliative care declared by the professionals.

**Table 3:** Definitions and perceptions of neonatal palliative care.

	MFMs, n%	FCPs, n%	*P* value
**The implementation of palliative care signifies withholding certain curative care options in the neonatal period.**	56 (56.6)	63 (57.3)	NS
**Neonatal palliative care is care intended to provide end-of-life support for patients.**	90 (90.9)	72 (64.9)	< 0.001
**Provision of palliative care to a neonate is fundamentally analogous to that provided to other patients.**	59 (59.6)	47 (42.3)	0.013
**The legal framework for the provision of adult palliative care should be different than that of neonates.**	46 (51.7)	66 (60.0)	NS
**The term "palliative care" is appropriate to use for neonates.**	80 (81.6)	79 (71.2)	NS
**The provision of palliative care amounts to "concealed euthanasia".**	18 (18.2)	16 (14.3)	NS
**Palliative care plans can be considered as an "alternative" to TOP.**	86 (87.8)	80 (71.4)	0.004

Rate signifies the percentage of professionals who agreed with the statement made.

### Barriers to and Influences on Perinatal Palliative Care

The most significant factor contributing to the decision to continue with the pregnancy following the diagnosis of a lethal fetal abnormality was "*to respect parental freedom of choice (their autonomy)*", as reported by quitely over half of professionals (56.0%, n = 120). From an opposite perspective, in one fourth of cases (25.7%, n = 55), the least important factor was "*to achieve parental acceptance of the diagnosis of lethal abnormality in case of initial refusal* ". Tables 4 and 5 show the factors that influence the management of lethal fetal abnormalities (Table 4) and point out the obstacles that interfere with providing adequate information about palliative care at birth (Table 5). The notion of "*the presumed quality of the baby's life given its condition*" seems to have the most influence on the professionals' course of action (97.1%, n = 204). The "*degree of diagnostic certainty*" appears to be the most important part (98.1%, n = 207) in the information provided to the woman with regard to potential palliative management.

**Table 4:** Categorization of factors that influence perinatal professionals when managing cases of lethal fetal abnormality.

	Rank	MFMs (%),	FCPs (%),	*P* value
**The presumed quality of the baby's life given its condition**	1	99 (100.0)	105 (94.6)	0.031
**The religious values of the future parent(s)**	2	96 (96.0)	108 (97.3)	0.71
**The degree of diagnostic certainty**	3	97 (97.0)	106 (96.4)	1.000
**The couple's request for a particular type of management**	4	95 (96.9)	107 (96.4)	1.000
**The moral values of the future parent(s)**	5	93 (93.9)	108 (97.3)	0.312
**The antenatal information provided about the various management options, including PC**	6	96 (97.0)	99 (90.8)	0.087
**The understanding level of the future parent(s)**	7	92 (94.8)	99 (90.0)	0.297
**The level of evidence of the information given to the couple about the condition**	8	94 (94.0)	95 (87.2)	0.105
**The degree of confidence of the future parent(s) in the MCPD team**	9	88 (88.0)	88 (81.5)	0.193
**The presumed duration of the baby's survival given its condition**	10	87 (88.8)	88 (81.5)	0.144
**The extent of the team's experience in palliative care**	11	86 (87.8)	87 (78.4)	0.073
**The stage of pregnancy**	12	70 (71.4)	89 (81.7)	0.099
**The possibility of "sparing suffering" by performing TOP**	13	63 (63.6)	57 (51.8)	0.094
**The socio-economic and cultural context of the future parent(s)**	14	43 (43.0)	65 (59.1)	0.020

P-Value for Pearson’s χ^2^ testing independence between groups of perinatal professionals (maternal fetal medicine specialists, MFMs; fetal care pediatric specialists, FCPs) and the opinion toward the factors that influence LFA (initially collected from a 4-point scale, opinions were recoded in a binary variable: scores from 1 to 2 represented lack of influence and scores from 3 to 4 represented the influence of the factor).

**Table 5:** Categorization of items influencing antenatal information about palliative care in cases of lethal fetal abnormality.

	Rank	MFMs (%),	FCPs (%),	*P* value
**The degree of diagnostic certainty**	1	96 (97.0)	111 (99.1)	0.256
**The couple's request concerning management**	2	97 (98.0)	105 (93.8)	0.129
**The quality of the relationship between antenatal and postnatal teams**	3	93 (93.0)	102 (91.9)	0.761
**The understanding level of the future parents**	4	94 (93.1)	100 (90.1)	0.437
**The experience and level of expertise of the team responsible for palliative care within the hospital**	5	93 (92.1)	97 (87.4)	0.263
**The possibility of suggesting a meeting with the neonatal palliative care team**	6	93 (93.0)	95 (86.4)	0.117
**The legal framework**	7	87 (87.0)	96 (85.7)	0.786
**The opinions of the MCPD group**	8	79 (78.2)	94 (83.9)	0.287
**The opinions of the antenatal care team on TOP and palliative care**	9	75 (74.3)	89 (80.2)	0.303
**The limitations and variability of perceptions of the care team**	10	68 (68.7)	85 (77.3)	0.162
**The socio-cultural characteristics of the future parents**	11	65 (65.0)	79 (71.2)	0.336
**The unease of care providers in dealing with death**	12	66 (66.0)	76 (67.9)	0.774

P-Value for Pearson’s χ^2^ testing independence between groups of perinatal professionals (maternal fetal medicine specialists, MFMs; fetal care pediatric specialists, FCPs) and the opinion toward the factors that influence antenatal communication of information about palliative care at birth in cases of lethal fetal abnormality (initially collected from a 4-point scale, opinions were recoded in a binary variable: scores from 1 to 2 represented lack of influence and scores from 3 to 4 represented the influence of the factor).

Professionals opinions were generally in favor (92.5%, n = 197, 94.1% and 91% among MFMs and FCPs respectively) of standardizing the practice of offering women the option of neonatal palliative care (in cases of lethal fetal abnormalities). Lastly, 64.6% (n = 135) of professionals supported, in certain circumstances, offering TOP without feticide with palliative care at birth.

## Discussion

Our study shows and accurately evaluates how LFA is defined and perceived by perinatal professionals, its impact on the information provided to parents (mainly regarding palliative care at birth), and the obstacles interfering with taking palliative care into consideration. In addition to this, our study was conducted on a non consensus opinion model at professional and national level. Moreover, it is the first study to evaluate the attitudes and opinions toward perinatal palliative care (PPC) in a national survey in France. It highlights the different factors (e.g., professional anticipations, uncertainty, psychosocial characteristics of parents) that affect the management of LFA in the French context. Its outcome can potentially be a first step toward creating a more global framework for PPC in France.

Our French national survey reveals the variation in perceptions and practices among MFMs and FCPs regarding the approach to pregnancy complicated by LFA. More specifically, it shows discrepancies in professional opinions of pediatric specialists concerning clinical, legal and ethical aspects of prenatal diagnosis of lethal fetal abnormality. This research satisfies the requirements for transparency set out by the Leonetti Law [14], supported by the French Neonatology Society [15]. It also satisfies the recommendations issued by national observatories and commissions on end-of-life and palliative care [16,17], and these recommendations underlined the need for a such research in this area, as already undertaken in other countries [18].

Even so, this study has its limitations. The limited participation rate could be explained by the length of questionnaire, but it is similar to the usual rate in qualitative pediatric studies [19]. Besides, there could be other reasons. We hypothesize that PPC, a relatively new area in neonatology, might conflict with personal convictions and the complexity of management of such pregnancies [12]. Some doctors also stated that their non-participation was due to a lack of knowledge in this area. It is recognized in the survey that the recruited sample does not represent the entire population but only a sample of people who agreed to participate in the study. Our data should be weighted due to intra-individual variability [20].

We report a paradoxical response rate concerning diagnostic and prognostic certainty in LFA. Ideally, PPC is offered when there is both diagnostic and prognostic certainty, but occasionally it is also offered in some cases of prognostic certainty with diagnostic uncertainty. Usually it is inappropriate to provide PPC when there is prognostic uncertainty, at least until neonatal examination and investigations are done [2]. Due to the fact that French law does not list the fetal abnormalities where TOP is allowed (however, it is almost impossible to have a such list due to continuous advances in knowledge), there is a considerable variability among physicians in assessing the legitimacy of pursuing the pregnancy. Even the term "lethal fetal abnormality" is controversial; "life-limiting condition" would be more appropriate [3]. Dommergues et al have assessed the cases of fetal abnormalities resulting in termination of pregnancy, and one of the aims of their study was to determine what degree of severity in such fetal conditions would result in TOP in France [21]. These authors considered lethal every condition in which the risk of perinatal or infant mortality has been estimated at over 90%, such as anencephaly. In response to this mentioned study and its outcome, Bétrémieux makes clear that 33% of the TOP described there were carried out in a context of lethal fetal condition. In France, there are around 8,000 terminations of pregnancy per year; so there are 2,500 terminations due to lethal fetal abnormality [22]. In 2012, 810 women chose to continue with their pregnancy in spite of the diagnosis of severe fetal abnormality to which, if ever requested, TOP could have been performed [23].

In this context, we think that it has become essential to develop, like in many other countries, a framework or guidelines for PPC. In the UK, the National Screening Committee provided guidance on the proportion of cases of structural and chromosomal abnormalities that it expects to detect (NHS 2010), and the International Society of Ultrasound in Obstetrics and Gynecology provides guidelines on the conduct of both screening and more targeted fetal ultrasound examinations in order to detect structural fetal anomalies [24]. Most of lethal structural abnormalities may be detected at 18–22 weeks. In this context, the British Association of Perinatal Medicine developed a Framework for PPC [8]. It indicates some of the clinical scenarios in which eligibility for palliative care could be considered. Our study reports that the level of diagnostic certainty is the most important criteria guiding the information provided to parents concerning potential need for neonatal palliative care. However, professionals cannot avoid the uncertainty due to limitations in the antenatal examination, the latter being fundamental for making a certain diagnosis and a reliable prognosis prior to birth. The women (or couples) also need to be informed about the limits of medical knowledge in general and the prenatal diagnosis in particular. Most of prenatal diagnoses that are deemed lethal need to be confirmed at birth [25].

Palliative care (as defined by French law [13]) is perceived differently by professionals. Less than half of the surveyed professionals had received practical training in palliative care and one quarter had received theoretical formation. Yet, in France, more than half of neonatal deaths followed decisions to limit and/or cease active treatment, and, therefore, fell under the category of palliative care [26]. The majority of professionals (less among FCPs) consider neonatal palliative care as care intended to provide end-of-life support for patients, and it can be considered as an "alternative" to TOP. The core intention of providing postnatal palliative care where the infant is brought to life surely differs from that of TOP where the fetus is to die.. Therefore, palliative support for a newborn can never simply be considered as an option or an alternative to TOP. It is not a matter of choosing between two different treatments for the same disease but choosing between two different intentions, belonging to two different episodes of time. The provision of palliative care is of an open and uncertain duration during which the well-being of the infant and their family is the priority; unlike TOP, which is "faster", "drastic", and performed according to a protocol [11].

This study highlighted the difficulties and discrepancies related to PPC in various domains of perinatal care. Notably, the difficulties associated with defining and diagnosing LFA remain an obstacle to the provision of information (in terms of both content and method of communication) and the implementation of PPC. In an antenatal context, identifying "unreasonable obstinacy" (or "futility"), the first step in implementing palliative care [27], is therefore problematic, with the concept often appearing vague or prospective (the anticipation of unreasonable obstinacy of treatment in a neonatal context). In other words, prognostic (and/or scientific) uncertainty constitutes an important factor in terms of choice of the approach and it can strengthen the possibility of choosing the first option: TOP. Resorting to TOP, in this context, could be considered as a norm, a norm strongly induced by uncertainty [12]. TOP without feticide drug prior to 24 weeks is another option, an option that enables the woman to avoid participating in fetal death and, sometimes, allowing her to meet her baby alive in the delivery room where palliative care is implemented [28]. Conversely, if the woman or couple is more tolerant toward this medical uncertainty, they will more likely continue with the pregnancy. Medical uncertainty allows them to fantasize that the child might be healthy at birth and that after all the diagnosis could be a mistake. The future parents may believe in the idea of "love that conquers all", and feel the need to prove it to the medical team. This uncertainty must be incorporated by the perinatal professionals in their provision of care; they must share it, accept it, understand its risks, and accordingly develop care strategies in collaboration with the woman or the couple. While this uncertainty is perceived as a limiting factor by medical team, it is actually the power that drives the woman or couple to continue with the pregnancy. Thus parents report a positive experience with PPC programs [29] where a specific detailed plan is provided determining the values and objectives of this type of care, recognizing the needs of both the patient and their family, and ensuring the presence of a multidisciplinary team trained in PPC working in an environment that is physically and environmentally adapted to meet these needs [30]. Furthermore, it would be useful to evaluate women’s motivation when taking their decision after announcement of the diagnosis. We need to find out if the variation of attitudes and perceptions among perinatal professionals would affect women's decisions about the pregnancy [31], and specially their decisions about termination of pregnancy due to lethal abnormality. In addition, this study demonstrates a clear need for providing PPC training for professionals and for standardizing PPC practices, and this would be facilitated by creating a PPC framework in France as it already exists in UK [8,9]. According to our outcomes, the woman's autonomy and freedom of choice are important concepts. Agreeing with this, the American College of Obstetricians and Gynecologists-Committee on Ethics and the American Academy of Pediatrics-Committee on Bioethics [10], along with the British Association of Perinatal Medicine [8] have all made recommendations for physicians dealing with families who are expecting a baby with lethal hereditary malformation, and they also focused on the importance of discussion: "The informed consent process should involve thorough discussion of the risks and benefits for both the fetus and the pregnant woman. The full range of options, including fetal intervention, postnatal therapy, palliative care, or pregnancy termination, should be discussed". French guidelines also contributed to the content of this discussion [6,14].

Eventually, consent (which is directly attributed to the information provided), although resulting from a legal obligation, raises some pertinent questions. How much freedom does the woman really have in choosing? Isn’t the situation itself imposing an inevitable decision? Is free choice possible given the pressure upon her (or the future parents) from medical professionals and/or society? In conclusion, given that 92.5% of the investigated perinatal professionals agreed with always informing the women about the option of neonatal palliative care in cases of lethal fetal abnormality, it is therefore essential to discuss and provide a framework for implementation of PPC.

## Supporting Information

S1 Questionnaire PPC Survey. (PDF)
