# Supplementary material for: Barriers in Referring Neonatal Patients to Perinatal Palliative Care: A French Multicenter Survey
Source: PLoS One. 2015 May 15;10(5):e0126861. doi: 10.1371/journal.pone.0126861 (PMC4433103; doi:10.1371/journal.pone.0126861)
Supplement: S1 Questionnaire — (PDF) [file pone.0126861.s001.pdf]

Étude PNSP
Perinatal Palliative Care Survey / Diagnostic prénatal de pathologie létale : information sur la prise en charge palliative néonatale

Numéro d'indentification /__/__/__/

DONNEES SOCIO-DEMOGRAPHIQUES

(1) Votre âge : /___/ ans
(2) Votre sexe

- ☐ 1 Homme
- ☐ 2 Femme

(3) Votre profession

- ☐ 1 Sage-femme
- ☐ 2 Psychologue
- ☐ 3 Médecin (si nécessaire cochez plusieurs cases)
  - ☐ 3.1 Néonatologie
  - ☐ 3.2 Réanimation pédiatrique
  - ☐ 3.3 Génétique
  - ☐ 3.4 Gynécologie Obstétrique
  - ☐ 3.5 Autres spécialités, précisez : …………………………………………

(4) Nombre d'années d'exercice de votre profession: /___/ ans
(5) Dans combien de centre pluridisciplinaire de diagnostic prénatal (CPDPN) avez-vous déjà exercé et combien d'année pour chacun? (Ex : 1 centre : 6 mois)

PREMIERE PARTIE : OPINIONS

(1) Citez spontanément les 3 premiers mots ou expressions qui vous viennent à l'esprit lorsqu'on vous parle de **pathologies fœtales létales.**
(2) Disposez-vous d'une **<u>liste</u>** de pathologies fœtales létales dans votre CPDPN ?

☐ 1 Oui ☐ 2 Non ☐ 3 NSP

(3) Pensez-vous qu'une telle liste est (ou serait) **utile** à la pratique d'un CPDPN ?

☐ 1	☐ 2	☐ 3	☐ 4
Non pas du tout	Plutôt non	Plutôt oui	Oui tout à fait

(4) Selon vous, quelle est la **finalité** d'un diagnostic anténatal ?

a. ***Diagnostiquer des pathologies de pronostic sévère chez le fœtus***

☐ 1	☐ 2	☐ 3	☐ 4
Non pas du tout	Plutôt non	Plutôt oui	Oui tout à fait

b. ***Evaluer l'état de santé du fœtus***

☐ 1	☐ 2	☐ 3	☐ 4
Non pas du tout	Plutôt non	Plutôt oui	Oui tout à fait

c. ***Envisager un projet de soin curatif en cas de pathologie***

☐ 1	☐ 2	☐ 3	☐ 4
Non pas du tout	Plutôt non	Plutôt oui	Oui tout à fait

d. ***Avoir pour but un rôle consultatif et d'information auprès des parents:***

❐1	❐2	❐3	❐4
Non pas du tout	Plutôt non	Plutôt oui	Oui tout à fait

(5) Pour chaque proposition concernant le diagnostic de pathologie fœtale létale, cochez la case qui correspond le mieux à ce que vous pensez.
Etablir un diagnostic de pathologie fœtale létale vous semble ?

❐1	❐2	❐3	❐4	❐5	❐6
Très difficile	***Difficile***	***Plutôt difficile***	***Plutôt facile***	***Facile***	***Très facile***

Dans le cadre d'une pathologie fœtale létale, le pronostic est…

❐1	❐2	❐3	❐4	❐5	❐6
Très incertain	***Incertain***	***Plutôt certain***	***Plutôt certain***	***Certain***	***Très certain***

(6) Selon vous, quelle proportion de cas de pathologies fœtales, présentées au CPDPN, pourrait correspondre à un diagnostic « qualifié » de pathologie fœtale létale ? /_ _ _ / **%**
(7) Selon vous, dans quelle mesure peut-on parler de pathologie fœtale létale ? Cochez la case qui correspond le mieux à ce que vous pensez.

a. ***Décès du nouveau-né à court terme***

❐1	❐2	❐3	❐4
Non pas du tout	Plutôt non	Plutôt oui	Oui tout à fait

b. ***Certitude d'une mort néonatale***

❐1	❐2	❐3	❐4
Non pas du tout	Plutôt non	Plutôt oui	Oui tout à fait

c. ***Aucun recours thérapeutique raisonnablement envisageable***

❐1	❐2	❐3	❐4
Non pas du tout	Plutôt non	Plutôt oui	Oui tout à fait

(8) Voici une « liste » de pathologies dans le tableau suivant. Indiquez pour chacune d'entre elles si, selon vous, une **prise en charge palliative** (SP) et/ou une **interruption médicale de grossesse** (IMG) et/ou un **traitement curatif** paraît le plus approprié. Pour chaque pathologie, cochez la ou les réponse(s) qui correspondent à votre opinion. *(Ne rien cocher si ne sait pas)*

	**SP**	**IMG**	**Traitement curatif**
a. ***Cardiopathie sévère inopérable***	❐1	❐2	❐3
b. ***Trisomie 18***	❐1	❐2	❐3
c. ***Trisomie 15***	❐1	❐2	❐3
d. ***Trisomie 13***	❐1	❐2	❐3
e. ***Hernie diaphragmatique majeure ou syndromique***	❐1	❐2	❐3
f. ***Anencéphalie***	❐1	❐2	❐3

g. ***Hydrocéphalie majeure***	❐1	❐2	❐3
h. ***Agénésie rénale bilatérale, syndrome de Potter***	❐1	❐2	❐3
i. ***Jumeaux siamois***	❐1	❐2	❐3
j. ***Exencéphalie***	❐1	❐2	❐3
k. ***Prématurité < 23SA***	❐1	❐2	❐3
l. ***Triploïdie***	❐1	❐2	❐3

(9) Voici une liste de **critères** qui pourraient avoir une **influence sur <u>l'orientation</u> de la prise en charge (IMG/SP)** en cas de pathologie fœtale létale. Pour chacun des critères nous vous demandons d'évaluer son niveau d'influence potentielle.

	Pas d'influence	***Influence faible***	***Influence moyenne***	***Influence forte***
a. Les valeurs morales du (ou des) parent(s).	❐1	❐2	❐3	❐4
b. Les valeurs religieuses du (ou des) parent(s).	❐1	❐2	❐3	❐4
c. Le stade d'avancement de la grossesse.	❐1	❐2	❐3	❐4
d. Le contexte socio-économique et culturel du (ou des) parent(s).	❐1	❐2	❐3	❐4
e. Le niveau de certitude du diagnostic.	❐1	❐2	❐3	❐4
f. Le niveau de preuve de l'information donnée au couple concernant la pathologie.	❐1	❐2	❐3	❐4
g. Le niveau de compréhension perçu du (ou des) parent(s).	❐1	❐2	❐3	❐4
h. L'information donnée en anténatal sur les différentes possibilités de prises en charge dont les SP.	❐1	❐2	❐3	❐4
i. Le niveau d'expérience de l'équipe en soins palliatifs.	❐1	❐2	❐3	❐4

j. La qualité de vie présumée du bébé au regard de sa pathologie	❐1	❐2	❐3	❐4
k. La durée de survie présumée du bébé par rapport à sa pathologie.	❐1	❐2	❐3	❐4
l. La demande du couple concernant un type de prise en charge.	❐1	❐2	❐3	❐4
m. La possibilité d'une « économie de souffrance » par la réalisation d'une IMG.	❐1	❐2	❐3	❐4
n. le degré de confiance du ou des parent(s) dans l'équipe du CPDPN.	❐1	❐2	❐3	❐4

(10) Vous trouverez ci-dessous quatre raisons qui peuvent contribuer **au choix d'une poursuite de grossesse** en cas de diagnostic de pathologie fœtale létale. Nous vous demandons de classer ces quatre raisons par ordre d'importance, de (1) la raison la plus importante à (4) la raison la moins importante.

/____/ Assurer, construire ou renforcer la parentalité

/____/ Respecter la liberté de choix parental (leur autonomie)

/____/ Diminuer la culpabilité d'un fœticide en cas d'IMG

/____/ Conduire à une acceptation parentale du diagnostic de pathologie létale en cas de déni initial

(11) Pouvez-vous indiquer la (ou les) autre(s) raison(s) qui fait (font) qu'une prise en charge palliative néonatale en cas de pathologie fœtale létale ne serait pas proposée chaque fois qu'elle serait envisageable ?

(12) Voici un certain nombre de propositions qui concernent la prise en charge palliative en médecine périnatale. Pour chacune d'entre elles, nous vous demandons de cocher la case qui correspond le mieux à ce que vous pensez.

a. ***La mise en œuvre des soins palliatifs marque l'absence de mise en œuvre de certains soins curatifs en période néonatale.***

❐1	❐2	❐3	❐4
Pas du tout d'accord	Plutôt pas d'accord	Plutôt d'accord	Tout à fait d'accord

b. ***Les soins palliatifs en période néonatale sont des soins qui ont pour but d'accompagner les patients vers la mort.***

❐1	❐2	❐3	❐4
Pas du tout d'accord	Plutôt pas d'accord	Plutôt d'accord	Tout à fait d'accord

c. ***La démarche palliative chez un nouveau né est fondamentalement analogue à celle d'un autre patient.***

❐1	❐2	❐3	❐4
Pas du tout d'accord	Plutôt pas d'accord	Plutôt d'accord	Tout à fait d'accord

d. ***Dans votre CPDPN, est-ce qu'une prise en charge psychologique est proposée au parents ?***

☐ 1 oui ☐ 2 non

e. ***Le soutien psychologique devrait être obligatoirement proposé pour les parents en cas de soins palliatifs de leur enfant.***

☐ 1 Pas du tout d'accord ☐ 2 Plutôt pas d'accord ☐ 3 Plutôt d'accord ☐ 4 Tout à fait d'accord

f. ***Le cadre légal (Loi Léonetti) de la démarche palliative adulte devrait être différent pour les soins palliatifs réalisés en période néonatale.***

☐ 1 Pas du tout d'accord ☐ 2 Plutôt pas d'accord ☐ 3 Plutôt d'accord ☐ 4 Tout à fait d'accord

g. ***Concernant les nouveau-nés, le terme de « soins palliatifs » est approprié.***

☐ 1 Pas du tout d'accord ☐ 2 Plutôt pas d'accord ☐ 3 Plutôt d'accord ☐ 4 Tout à fait d'accord

h. ***La démarche de soins palliatifs revient à faire une euthanasie déguisée.***

☐ 1 Pas du tout d'accord ☐ 2 Plutôt pas d'accord ☐ 3 Plutôt d'accord ☐ 4 Tout à fait d'accord

i. ***Les projets de naissance en soins palliatifs peuvent être considérés comme une « alternative » (définie comme une solution de remplacement) à l'IMG.***

☐ 1 Pas du tout d'accord ☐ 2 Plutôt pas d'accord ☐ 3 Plutôt d'accord ☐ 4 Tout à fait d'accord

DEUXIEME PARTIE : PRATIQUES

(13) Avez-vous reçu une formation sur les soins palliatifs ?

☐ 1 Formation dans le cadre de l'enseignement supérieur (Master, DU, DIU, CEU,…)
☐ 2 Modules d'enseignement dans le cadre de votre formation continue
☐ 3 Autres, précisez : ………………………………..
☐ 4 Pas de formation théorique
☐ 5 Formation pratique, précisez : ………………………………..

(14) Avez-vous le sentiment de réaliser des « soins palliatifs » (définition Loi juin 1999* et article L. 1110-9 du Code de la Santé Publique, www.legifrance.gouv.fr) dans le cadre d'une prise en charge postnatale de pathologies létales?

** « des soins actifs et continus pratiqués par une équipe interdisciplinaire en institution ou à domicile. Ils visent à soulager la douleur, apaiser la souffrance psychique, à sauvegarder la dignité de la personne malade et à soutenir son entourage. »*

☐ 1	☐ 2	☐ 3	☐ 4
Non pas du tout	Plutôt non	Plutôt oui	Oui tout à fait

(15) Selon vous, quelle proportion de cas de pathologies fœtales, présentées au CPDPN, pourrait faire l'objet d'une prise en charge palliative à la naissance ? **/_ _ _ / %**

(16) Citez les 3 mots ou expressions qui vous viennent spontanément à l'esprit lorsqu'on vous parle de **soins palliatifs néonatals.**

(17) Les questions qui suivent concernent **les deux dernières patientes** que vous avez suivies et pour lesquelles un diagnostic de pathologie fœtale létale a été posé. Si vous n'êtes pas en mesure de vous prononcer car vous n'êtes pas concerné par la question nous vous demandons de cocher la réponse « Non concerné ».

	**DOSSIER N°1**	**DOSSIER N°2**
a. ***Diagnostic évoqué :***	………………	………………
b. ***Avez-vous <u>expliqué personnellement</u> le diagnostic à la patiente?***	☐ 1 Oui<br>☐ 2 Non<br>☐ 3 Non concerné	☐ 4 Oui<br>☐ 5 Non<br>☐ 6 Non concerné
c. ***Selon vous, l'information a-t-elle été donnée de manière pluridisciplinaire ?***	☐ 1 Oui<br>☐ 2 Non<br>☐ 3 Non concerné	☐ 4 Oui<br>☐ 5 Non<br>☐ 6 Non concerné
d. ***Avez-vous, <u>spontanément</u>, informé (sans demande de leur part) des possibilités de prise en charge en cas de naissance ?***	☐ 1 Oui<br>☐ 2 Non<br>☐ 3 Non concerné	☐ 4 Oui<br>☐ 5 Non<br>☐ 6 Non concerné
e. ***Vous a-t-elle formulé <u>spontanément</u> une demande d'IMG ?***	☐ 1 Oui<br>☐ 2 Non<br>☐ 3 Non concerné	☐ 4 Oui<br>☐ 5 Non<br>☐ 6 Non concerné
f. ***Vous a-t-elle formulé <u>spontanément</u> une demande de poursuite de grossesse ?***	☐ 1 Oui<br>☐ 2 Non<br>☐ 3 Non concerné	☐ 4 Oui<br>☐ 5 Non<br>☐ 6 Non concerné
g. ***Avez-vous précisé <u>spontanément</u> le cadre légal (Loi Veil pour l'IMG ou Loi Léonetti relative aux droits des malades et à la fin de vie) dans ce contexte ?***	☐ 1 Oui<br>☐ 2 Non<br>☐ 3 Non concerné	☐ 4 Oui<br>☐ 5 Non<br>☐ 6 Non concerné

h. ***Avez-vous précisé <u>spontanément</u> les différents scénarii possibles en cas de poursuite de grossesse et de naissance ?***	☐ 1 Oui<br>☐ 2 Non<br>☐ 3 Non concerné	☐ 4 Oui<br>☐ 5 Non<br>☐ 6 Non concerné

(18) Voici une liste de critères qui pourraient avoir une influence sur **l'information anténatale** en cas de pathologie fœtale létale. Pour chacun des critères nous vous demandons d'évaluer son niveau d'influence potentielle.

	Pas d'influence	*Influence Faible*	*Influence moyenne*	*Influence Forte*
a. Le cadre légal	❒1	❒2	❒3	❒4
b. La demande du couple concernant une prise en charge	❒1	❒2	❒3	❒4
c. Les caractéristiques socio culturelles des parents	❒1	❒2	❒3	❒4
d. Le niveau de compréhension perçu des parents	❒1	❒2	❒3	❒4
e. Le niveau de certitude du diagnostic	❒1	❒2	❒3	❒4
f. L'expérience et le niveau d'expertise de l'équipe en charge des soins palliatifs dans l'hôpital	❒1	❒2	❒3	❒4
g. Les opinions de l'équipe de soins en anténatal concernant l'IMG et les soins palliatifs	❒1	❒2	❒3	❒4
h. Les opinions du groupe du CPDPN	❒1	❒2	❒3	❒4
i. La qualité des liens entre les équipes anté et postnatales	❒1	❒2	❒3	❒4
j. La possibilité de proposer un entretien avec l'équipe de soins palliatifs néonatals (ou l'équipe ressource régionale de soins palliatifs pédiatriques)	❒1	❒2	❒3	❒4
k. Les limites et les représentations de l'équipe soignante	❒1	❒2	❒3	❒4
l. Le malaise des soignants face à la mort	❒1	❒2	❒3	❒4

(19) Avez-vous (ou votre conjoint) vécu <u>personnellement</u> un décès périnatal et/ou un accompagnement en fin de vie ?

☐ 1 Non
☐ 2 Oui

(20) En quoi ce vécu a-t-il modifié vos pratiques professionnelles?

(21) A propos de **l'information systématique** auprès des parents de la possibilité d'une prise en charge palliative néonatale (en cas de pathologie fœtale létale), vous diriez que vous y êtes :

❒1	❒2	❒3	❒4
Pas du tout favorable	Plutôt pas favorable	Plutôt favorable	Très favorable

(22) Etes vous favorable aux soins palliatifs chez le nouveau-né pour cause malformative ?

❒1	❒2	❒3	❒4
Pas du tout favorable	Plutôt pas favorable	Plutôt favorable	Très favorable

(23) Pensez-vous qu'un membre de l'équipe ressource régionale de soins palliatifs pédiatriques (ERRSPP) devrait faire partie du CPDPN et assister aux réunions décisionnelles ?

❒1	❒2	❒3	❒4
Pas du tout favorable	Plutôt pas favorable	Plutôt favorable	Très favorable

(24) Seriez-vous prêt à envisager dans certains cas la possibilité d'IMG sans foeticide avec soins palliatifs à la naissance ?

❒1	❒2	❒3	❒4
Pas du tout favorable	Plutôt pas favorable	Plutôt favorable	Très favorable
